# Understanding Others' Regret: A *f*MRI Study

**DOI:** 10.1371/journal.pone.0007402

**Published:** 2009-10-14

**Authors:** Nicola Canessa, Matteo Motterlini, Cinzia Di Dio, Daniela Perani, Paola Scifo, Stefano F. Cappa, Giacomo Rizzolatti

**Affiliations:** 1 Centro di Ricerca in Epistemologia Sperimentale e Applicata (CRESA), Vita-Salute San Raffaele University, Milano, Italy; 2 Division of Neuroscience, San Raffaele Scientific Institute, Milano, Italy; 3 Cognitive Neuroscience Center (CNC), Vita-Salute San Raffaele University, Milano, Italy; 4 Nuclear Medicine Unit, San Raffaele Scientific Institute, Milano, Italy; 5 Department of Neuroscience, University of Parma, Parma, Italy; 6 National Institute of Neuroscience, Torino, Italy; Macquarie University, Australia

## Abstract

Previous studies showed that the understanding of others' basic emotional experiences is based on a “resonant” mechanism, i.e., on the reactivation, in the observer's brain, of the cerebral areas associated with those experiences. The present study aimed to investigate whether the same neural mechanism is activated both when experiencing and attending complex, cognitively-generated, emotions. A gambling task and functional-Magnetic-Resonance-Imaging (*f*MRI) were used to test this hypothesis using *regret*, the negative cognitively-based emotion resulting from an unfavorable counterfactual comparison between the outcomes of chosen and discarded options. Do the same brain structures that mediate the experience of regret become active in the observation of situations eliciting regret in another individual? Here we show that observing the regretful outcomes of someone else's choices activates the same regions that are activated during a first-person experience of regret, i.e. the ventromedial prefrontal cortex, anterior cingulate cortex and hippocampus. These results extend the possible role of a mirror-like mechanism beyond basic emotions.

## Introduction

From the early stages of cognitive development, humans are able to represent and understand others' mental and emotional states [1]. It has been suggested that the neural bases of this ability may rely on the mirror mechanism [2], [3]. The mirror mechanism has been investigated in two major domains, i.e. sensorimotor and emotional, involving two main circuits. One is located on the lateral convexity of the cortex, and includes the inferior parietal lobule (IPL) and the ventral premotor cortex plus the caudal part of the inferior frontal gyrus (IFG). This circuit mediates the understanding of gestures and meaningful actions [2], [3]. The second circuit, which includes the insula and anterior cingulate cortex (ACC), is involved in the experiential understanding of others' emotional states shaping interpersonal relations at a basic level [4]–[7].

Although there may be several ways in which others' emotions can be understood, recent studies indicate that one such mechanism is based on the reactivation of the cerebral areas associated with the observer's direct emotional experience [6]. Yet, neural mirror-responses have been assessed only in conditions involving basic-level emotional stimuli, such as visual expressions of disgust [5] or cues signaling pain [7]. As far as complex emotions are concerned, to date there is only behavioral evidence to suggest the involvement of a mirror-like mechanism in the automatic understanding of others' emotional states [8], [9].

To further advance our understanding of complex emotional processes, the present study investigates whether the understanding of others' negative emotions involves the activation of the same neural mechanism as in the first-person experience. Specifically, we investigated whether a neural resonance system is also engaged in situations involving complex emotions that emerge at the interface with high-level cognitive processing. To this purpose we used *regret*, a cognitively-based emotion that occurs when one's outcome is worse than the outcome one would have obtained had one made a different choice. Unlike basic emotions, regret stems from the counterfactual comparison between alternative outcomes, as when the chosen option in a gamble results in a negative outcome *compared with* that of the unselected alternative [10]. The possibility to quantify and evaluate the values associated with unselected alternatives, resulting in better outcomes than the one obtained, is crucial for regret to occur. Additionally, the emotion of regret is elicited when the individual feels a personal responsibility upon the outcome of her/his deliberate choice. Without these prerequisites, regret would be replaced by the basic emotion of disappointment.

Evidence that regret and disappointment are mediated by neural structures only partially overlapping comes from clinical [11] and brain imaging studies [12], that employed gambling to assess the neural underpinnings of these emotions. These studies showed that the experience of regret specifically involves the activation of the medial orbito frontal cortex (mOFC) [11], [12], ACC and hippocampus [12].

**Figure 1:**
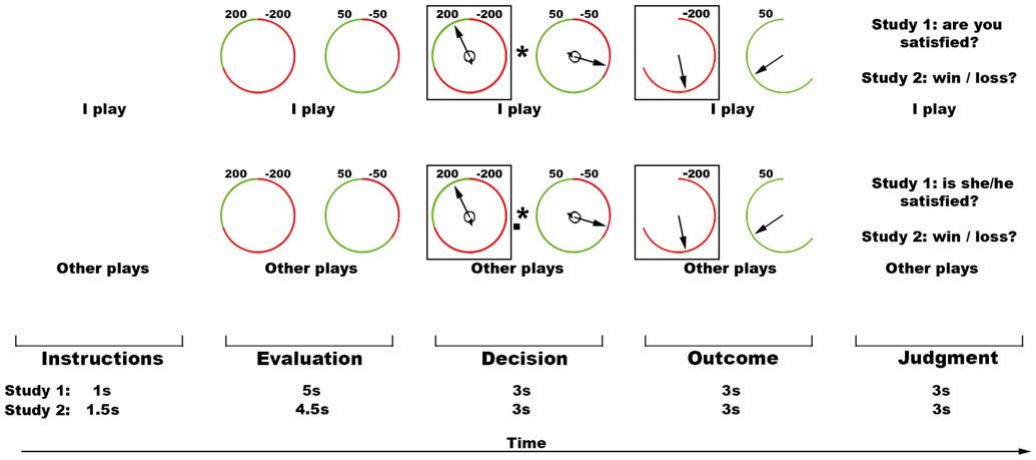
Experimental conditions, Studies 1 and 2. From left to right, schematic depiction of the sequence of events in the conditions IP (“I play”, top) and OP (“Other plays”, bottom). Within each condition there are 5 phases: instruction, evaluation of the wheels, choice of the gamble, outcome evaluation and judgment of the outcome. In the depicted example, the participant chose the loosing wheel. The length in seconds of each sub-event in the two studies is shown, in the inferior-most part of the figure.

In the present work, we extended the studies on regret by investigating whether the same cortical areas involved in the first person experience of regret become active also when the individual is faced with emotional experiences of regret in others. Two *f*MRI studies testing mirror-like responses to regret were carried out. In both studies, participants chose one of two gambles resulting in real wins or losses, like in previous investigations [11], [12]. Unlike previous works, though, in the present studies the participants also observed the same sequence of events (gambles evaluation, decision, outcome evaluation), this time experienced by another individual (see Figure 1).

As noted above, regret results from a sense of responsibility. Therefore, to address specifically regret, as opposed to disappointment, in two control conditions a computer program randomly chose one of the gambles for the participant or for the other player. In these instances, the computer choices still resulted in real monetary gains or losses for the players but, given the participants' lack of responsibility upon the gamble selection, the game outcome did not result in the feeling of regret [12].

The main difference between the two studies lies in the nature of the participants' task when presented with the outcomes obtained. In the first study, we ensured that the participants' emotional reaction to the results of the gambles was consistent with the actual counterfactual comparison between the obtained and unobtained outcomes (i.e., satisfied or unsatisfied with the outcome). In this way, we could also assess the participants' understanding of the other players' emotional state at outcome evaluation during “Other Plays” condition. More precisely, the participants were asked to indicate, after each trial, whether they were satisfied with their own decision (“I Play” condition) or whether, in their opinion, the other player was satisfied with her/his decision (“Other Plays” condition). Although this response was necessary to unfold the participants' emotional coherence with the actual outcomes in both IP and OP tasks, this type of judgment, by its nature, is likely to prompt an emotional response in the beholder. Since one requirement for a mirror response is its automaticity, to make sure that the observed activations were not affected by the explicit emotional appraisal of the gamble results, in the second study participants were required to give a non-emotional evaluation of the outcomes indicating whether results represented a win or loss.

Finally, to shed light on the question of whether the engagement of a resonance mechanism when attending someone else's experience of regret is affected by the individuals' empathic aptitude, we compared brain activations of females and males, under the assumption that females are more empathic than males [13].

## Results

### Study 1

The reported activations are based on the contrasts between the conditions where the players (the participant or the actor) made the decision *versus* the control conditions (IP *minus* IF; OP *minus* OF). These contrasts aimed at controlling for activations merely related to the carrying out of the tasks (e.g. visual, motor, etc.) and to highlight those underlying regret, i.e. outcome evaluation when one was responsible for her/his own choices. Behavioral measures confirmed that participants paid attention to the outcomes of all experimental conditions (see Text S1 for details).

In line with previous works on the neural correlates of regret processing [12], a *parametric* analysis was carried out to highlight the regions showing a positive linear relationship between regional signal change and the objective amount of regret in the condition “IP *minus* IF” or “OP *minus* OF”. Additionally, to investigate the possible involvement of a resonance-mapping system for regret, we focused on the *common* parametric effects across tasks, that is on the cerebral regions activated both when experiencing regret (IP *minus* IF) and when being aware of regret experienced by someone else (OP *minus* OF) (see Tables S1–[Supplementary-material pone.0007402.s003] S3 and Figure S1 for the description of the activated foci in the IP and OP tasks separately, as well as in the formal direct comparisons between them).

**Figure 2:**
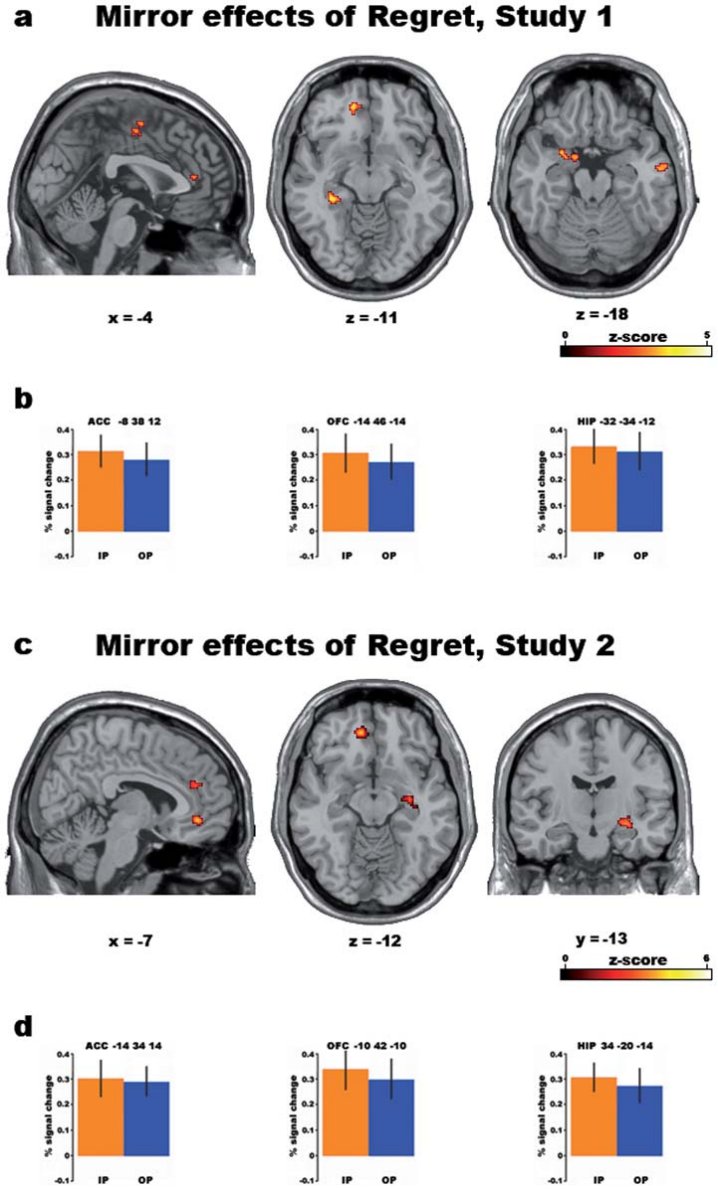
Common parametric effects of *regret* in Studies 1 and 2. Activations linearly and positively related to the objective amount of regret (measured as the difference between the outcomes of the chosen and unchosen gambles) in *both* the IP (*minus* IF) and OP (*minus* OF) conditions in Studies 1 and 2 (Conjunction analysis; p<0.001 uncorrected). a) Study 1: representative sections from the MNI305 template brain. From left to right: sagittal section showing activations in supplementary motor area (SMA), middle cingulate cortex and anterior cingulate cortex (ACC); horizontal section showing activations in ventromedial prefrontal cortex (vmPFC) and hippocampus (HIP); horizontal section showing left amygdala and right middle temporal gyrus activations. b) Study 1: from left to right, percent BOLD signal change (4 mm-radius sphere centered on the local maxima) in the Anterior Cingulate Cortex (ACC), ventromedial prefrontal cortex (vmPFC) and Hippocampus (HIP) is shown for both “I Play” (IP, yellow) and “Other Plays” (OP, blue) conditions. c) Study 2: representative sections from the MNI305 template brain. From left to right: sagittal section showing activations in middle cingulate cortex and ACC; horizontal section showing activations in vmPFC and HIP; coronal section showing right HIP activation. d) Study 2: from left to right, percent BOLD signal change in the same areas as in b).

The conjunction analysis between IP and OP statistical maps (relative to IF and OF, respectively; p<0.001 uncorrected) revealed significant common parametric activations in the left ventromedial prefrontal cortex (vmPFC), left amygdala and bilaterally in the hippocampus (Table 1, Figure 2a). Common parametric activations were also observed in the dorsal anterior cingulate cortex (ACC), and in a cluster extending from the supplementary motor area (SMA) to the middle cingulate cortex, as well as in the right middle temporal gyrus.

**Table 1:** Study 1, parametric analysis of *regret*: conjunction IP and OP conditions.

H	Anatomical region (BA)	MNI			Z-score
		x	y	z	
	**IP (** ***minus*** ** IF) and OP (** ***minus*** ** OF)**				
					
L	vmPFC (11)	−14	46	−14	3.62
L	Anterior cingulate cortex (24/32)	−8	38	12	3.31
R	Anterior cingulate cortex (24/32)	2	38	12	3.32
L	SMA (6)	0	−8	58	3.28
L/R	SMA (6)	4	−14	52	3.39
R	Middle cingulate cortex (6)	−4	−6	48	3.34
R	Middle temporal gyrus (21)	58	−10	−18	3.40
L	Amygdala	−14	−2	−18	3.26
L	Temporal pole (38)	−28	4	−20	3.47
	Amygdala	−24	0	−20	3.32
L	Hippocampus	−32	−34	−12	3.61
R	Hippocampus	32	−10	−32	3.53

Activations linearly and positively related to the objective amount of regret (measured as the difference between the actual outcome and the outcome of the unchosen gamble) in both the IP (*minus* IF) and OP (*minus* OF) conditions in study 1 (*p*<0.001 uncorrected). H = Hemisphere, L = Left, R = Right, BA = estimated Brodmann Area, vmPFC = ventromedial Prefrontal Cortex, SMA = Supplementary Motor Area.

**Figure 3:**
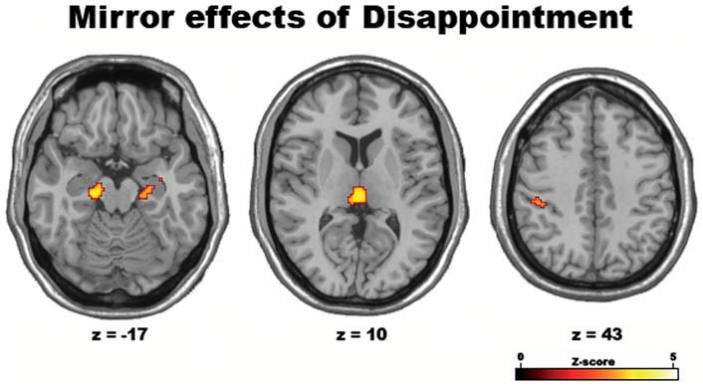
Common parametric effects of disappointment in Study 1. Shared effect of the parametric amount of disappointment (measured as the difference between the actual and unobtained outcome of the chosen gamble) across IP (*minus* IF) and OP (*minus* OF) conditions in Study 1, as shown by the results of a conjunction-analysis (p<0.001).

To make sure that these results did not only reflect an emotional response to a negative outcome *per se*, in a separate analysis we investigated the regions where activity was related to *disappointment* (i.e., win or loss in the chosen gamble, independent of the outcome of the unselected one). Common parametric activations to IP and OP tasks were observed in a number of areas including the left postcentral gyrus, the parahippocampal gyrus bilaterally, thalamus and brainstem periaqueductal grey matter (Table 2, Figure 3) but, crucially, in neither vmPFC nor ACC.

**Table 2:** Study 1, parametric analysis of *disappointment*: conjunction IP and OP conditions.

H	Anatomical region (BA)	MNI			Z-score
		x	y	z	
	**IP (** ***minus*** ** IF) and OP (** ***minus*** ** OF)**				
					
L	Postcentral gyrus (2)	−46	−30	42	3.20
R	Hippocampus	14	−28	−10	4.18
R	Hippocampus	26	−16	−18	3.33
L	Parahippocampal gyrus	−18	−22	−18	4.07
L/R	Thalamus/periacqueductal grey matter	0	−24	10	4.13
L/R	Cerebellum vermis 3	−2	−36	2	4.66
R	Cerebellum (VI)	30	−44	−26	3.67
L/R	Brainstem	−6	−20	−26	3.99

Activations linearly and positively related to the objective amount of disappointment (measured as the difference between the obtained and unobtained outcomes of the *chosen* gamble) in both the IP (*minus* IF) and OP (*minus* OF) conditions in study 1 (*p*<0.001 uncorrected). H = Hemisphere, L = Left, R = Right, BA = estimated Brodmann Area.

### Study 2

Like in study 1, here we carried out a conjunction analysis of the parametric effects observed between IP (*minus* IF) and OP (*minus* OF) conditions. This analysis confirmed the results of study 1, in that mirror-like effects were found in the left ventromedial PFC and dorsal anterior cingulate cortex (Table 3, Figure 2b). As far as hippocampal activation is concerned, in study 2 we found a stronger activation in the right hemisphere, as opposed to an enhanced activation observed in the left hemisphere in study 1. However, these results are not in conflict since, as it can be observed from Figure S1, a parametric effect of regret was observed in the right hippocampus in both IP and OP conditions also in study 1, though the respective foci did not overlap. Finally, a few differences were observed with respect to study 1, the most notable being a lack of activation of the left amygdala.

**Table 3:** Study 2, parametric analysis of *regret*: conjunction IP and OP conditions.

H	Anatomical region (BA)	MNI			Z-score
		x	y	z	
	**IP (** ***minus*** ** IF) and OP (** ***minus*** ** OF)**				
L	vmPFC (11/10)	−10	42	−10	4.26
L	Lateral OFC/anterior insula (11/38)	−26	16	−20	3.98
L	Anterior cingulate cortex (24/32)	−14	34	14	3.28
R	Middle cingulate cortex (24)	12	4	22	3.93
R	Middle frontal gyrus (46)	46	44	18	3.80
	Inferior frontal gyrus (45)	50	40	16	3.58
R	Amygdala	28	−12	−12	3.28
	Hippocampus	34	−20	−14	3.36
R	Dorsal striatum	12	14	8	3.64

Activations linearly and positively related to the objective amount of regret in both the IP (*minus* IF) and OP (*minus* OF) conditions in study 2 (*p*<0.001 uncorrected). H = Hemisphere, L = Left, R = Right, BA = estimated Brodmann Area, vmPFC = ventromedial Prefrontal Cortex, OFC = OrbitoFrontal Cortex.

### Individual Empathy-Scores and Gender Effects

During a post-scanning session, participants had to complete an Italian translation [14] of the Balanced-Emotional-Empathy-Scale (BEES; [15]), a test assessing emotional empathy.

Behavioral data from the BEES showed that the mean scores for our participants in study 1 were 34.83 (s.d. = 16.75) for females and 19.33 (s.d. = 18.39) for males. These data were representative of the normal Italian population (female mean = 37, s.d. = 18; male mean = 21, s.d. = 18; [14]) and revealed a significant gender difference, females being more empathic than males (Kolmogorov-Smirnov test for normality: *d* = 0.091, *p*>0.2; two-sample t-test, N = 24, *t*(22) = 2.15, *p* = 0.042).

**Figure 4:**
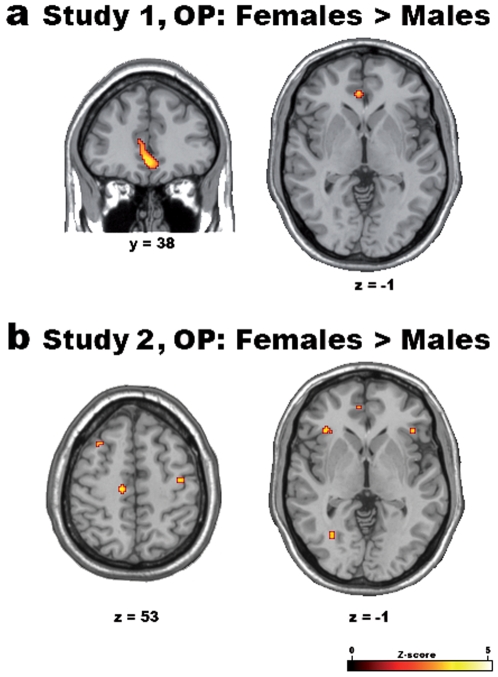
Differential parametric effects of gender on attended regret in the “Other Plays” (OP) condition. The different linear parametric effect of regret for female *vs*. male participants in Studies 1 (a) and 2 (b) (thresholded at p<0.001 uncorrected) in the OP (*minus* OF) condition are shown on 3D-renderings and representative slices of the MNI305 template brain.

Consistent with these results, direct gender comparisons carried out in the parametric statistical maps of the third-person task (OP *minus* OF) revealed stronger activations for females than males in the ventromedial PFC, in ACC and in portions of the parietal cortex bilaterally, including the somatosensory cortex and the inferior parietal lobule (Table 4, Figure 4a).

**Table 4:** Study 1, parametric analysis of *regret*: direct gender-comparisons in OP condition.

H	Anatomical region (BA)	MNI			Z-score
		x	y	z	
	**a. OP ** ***minus*** ** OF: Females>Males**				
					
L	vmPFC (11)	−4	40	−8	4.36
	Anterior cingulate cortex (11/32)	−12	42	14	3.43
L	Supramarginal gyrus (2/40)	−56	−34	36	5.18
	Inferior parietal lobule (2)	−56	−30	42	4.08
R	Postcentral gyrus (2/1)	44	−40	58	4.55
	Inferior parietal lobule (2)	54	−40	56	3.93
					
	**b. OP ** ***minus*** ** OF: Males>Females**				
					
L	Hippocampus	−40	−18	−16	4.75
L	Hippocampus	−28	−14	−32	3.86
R	Hippocampus	40	−22	−12	4.63
R	Hippocampus	30	−12	−26	5.02

Cerebral regions showing significant gender-effects related to the objective amount of attended regret in the OP (*minus* OF) condition in study 1 (*p*<0.001 uncorrected). H = Hemisphere, L = Left, R = Right, BA = estimated Brodmann Area, vmPFC = ventromedial Prefrontal Cortex.

These findings were confirmed in OP condition (*minus* OF) of study 2, where enhanced activations for females with respect to males were observed in the ventromedial PFC and somatosensory cortex bilaterally (Table 5, Figure 4b). However, unlike study 1, an enhanced activation for females was also observed in the anterior insula bilaterally (Figure 4b). This result can be interpreted in relation to the behavioral scores obtained on the BEES in study 2, that not only showed a higher mean difference between females and males than that observed in study 1, but also higher scores for females with respect to those obtained by their peers from study 1 (females' mean = 53.83, s.d. = 11.37; males' mean = 23.08, s.d. = 27.11; Kolmogorov-Smirnov test for normality: *d* = 0.19, p>0.2; two-sample t-test, N = 24, *t*(22) = 3.62, *p* = 0.007).

**Table 5:** Study 2, parametric analysis of *regret*: direct gender-comparisons in OP condition.

H	Anatomical region (BA)	MNI			Z-score
		x	y	z	
	**a. OP ** ***minus*** ** OF: Females>Males**				
					
L/R	ACC/vmPFC (10/11)	−6	48	0	3.57
L	Medial OFC (11)	−10	54	−14	3.36
L	Anterior insula/IFG pars orbitalis (47)	−34	28	−4	3.69
R	Anterior insula/IFG pars orbitalis (47)	42	28	−2	3.36
L	SMA/Middle cingulate cortex (6)	−14	−22	52	3.53
L	Postcentral gyrus (3b)	−18	−40	58	3.42
R	Sensorimotor cortex (4a/1)	40	−14	52	3.41
					
	**b. OP ** ***minus*** ** OF: Males>Females**				
					
L/R	Middle cingulate cortex (23)	−8	14	28	3.96
L/R	Superior medial gyrus (9)	−4	52	36	3.34

Cerebral regions showing significant gender-effects related to the objective amount of attended regret in the OP (*minus* OF) condition in study 2 (*p*<0.001 uncorrected). H = Hemisphere, L = Left, R = Right, BA = estimated Brodmann Area, ACC = Anterior Cingulate Cortex, vmPFC = ventromedial Prefrontal Cortex, OFC = OrbitoFrontal Cortex, IFG = Inferior frontal gyrus, SMA = Supplementary Motor Area.

## Discussion

The aim of the present study was to investigate whether the understanding of complex emotions, like regret, in others involves the reactivation of the cerebral areas associated with the observer's direct emotional experience. Regret is a negative emotion arising from a counterfactual comparison between the outcome of chosen and discarded options, whereby the discarded option would have produced higher benefits to the individual [10]. Regret thus requires two conditions to occur: namely, the feeling of responsibility for the decision made and a post-decisional evaluation of possible unselected alternatives associated with better outcomes than the one obtained. These two conditions define the emotional and cognitive differences underpinning regret with respect to other negative emotions like disappointment for a loss [12].

In this study we controlled for the effect of regret on cerebral activity by means of methodological and statistical measures. Methodologically, we dealt with the players' feeling of responsibility by comparing the conditions in which the participants actively made a deliberate choice (IP, OP) with control conditions in which choices were randomly made by the computer (IF, OF). Statistically, we used a parametric analysis to investigate only those areas whose activity showed a positive relation with increasing levels of regret. Specifically, we modeled the difference between the outcome of the chosen and unchosen gambles, so that also positive outcomes could result in regret if compared to an even more positive unselected outcome. Violation to these assumptions (feeling of the responsibility and counterfactual evaluation) lead to another emotional state, namely disappointment, even when faced with the same amount of loss.

The neural correlates of regret processing have been previously investigated using *f*MRI. These studies, carried out on healthy volunteers playing a gambling task similar to that employed in the present experiments, showed that the experience of regret is associated with the activation of OFC alongside structures involved in cognitively-induced responses to aversive and painful stimuli (ACC), and in declarative memory (hippocampal regions) [12] (see also [11] below).

What distinguishes the present study from the previous ones is a specific focus to the understanding of the experience of regret when observing someone else experiencing it, i.e. a resonance mirror effect that, to date, has been investigated only with basic-level emotional stimuli. Among the studies addressing mirroring in the emotional system, of particular interest is the *f*MRI study by Singer *et al.* [7], where volunteers either experienced a painful stimulus or observed a cue indicating that their loved one, present in the same room, was receiving a similar stimulation. The areas that were activated both when the volunteers were experiencing pain and when they knew that the other individual was experiencing it, were the anterior insula bilaterally and the ACC. Similar results were reported also for disgust. As for pain, feeling disgust or observing someone expressing it activates the anterior insula and the ACC [5].

In line with these studies [5], [7] (see also [16], [17] and [6] for a review), we focused on the common effects observed in the cerebral regions that were activated both when experiencing regret (IP *minus* IF) and when observing the regretful outcome of another player (OP *minus* OF).

Our data on the parametric effects common to IP and OP tasks (relative to baseline) in both studies 1 and 2 revealed several activation foci including the ventromedial prefrontal cortex, the dorsal anterior cingulate cortex (ACC) and hippocampus (Tables 1 and 3, Figure 2). These results confirm previous findings [12], [18] and, crucially, show that the activation of these regions also occurs when participants observe the other player's regretful outcomes. It is worth noting that the results from study 1 revealed a modulation of activity also in the amygdala that was not confirmed in our second study. In this respect, it is likely that, in study 1, amygdala activation was enhanced by the emotional nature of the judgment provided by the participants, and lack of activation in study 2 shows that modulation of its activity is not specific for regret. This lack of emotion-specificity is in contrast with vmPFC activation that, on the other hand, is core to the expression of regret.

Largely on the basis of evidence coming from animal studies, the *medial* portion of ventral prefrontal cortex is thought to be associated with positive reward processing, as opposed to its *lateral* part that instead is supposed to be involved in the processing of negative stimulus valence [19]. However, several studies have highlighted a more complex picture, according to which the medial portion of ventral prefrontal cortex is engaged in the processing of both positive and negative emotional events [20]. What the present and previous works strongly suggest, however, is that not all types of emotion are associated with vmPFC activation; rather there seems to be a specific involvement of this area in the processing of complex emotions. A convincing evidence in this respect comes from clinical studies, showing that patients with medial PFC lesions that performed a gambling task similar to that employed in this study could not process the emotion of regret elicited by the counterfactual comparison between the selected outcome and those of unselected alternatives [11]. Notably, however, those patients could exhibit emotional arousal to a loss when the observation of post-decisional outcome did not induce any counterfactual reasoning, i.e. disappointment.

These results confirm the view that vmPFC defines the emotional value of the error given by the difference between the obtained outcome and the unselected alternatives that, if chosen, would have produced better results. This error, which emotionally results in the negative feeling of regret, is a necessary drive for behavioral reorganization. Anterior cingulate cortex uses information about the emotional valence of unsuccessful behavior to re-organize future choices accordingly [21]. In other words, the negative emotion associated with regret is the basis of the motivation to workout alternative solutions in response to the reoccurrence of future similar situations. This motivation lacks in disappointment, where the individual has no feeling of responsibility upon the outcome and is powerless with respect to her/his loss.

Core of this study are the common effects observed between the conditions IP and OP (after baseline subtraction), which indicate that vmPFC-ACC and hippocampal activations mediate the processing of regret not only when directly experienced, but also when knowing that someone else is facing a counterfactual negative outcome. More specifically, this finding shows that the understanding of others' regret is mediated by the reactivation of the same core cerebral regions that induce the feeling of regret in the beholder during a first person experience, hence supporting the involvement of a resonance, mirror-like, mechanism in the comprehension of the high-order emotion of regret when experienced by others. Through this mechanism, others' emotional states are mapped onto the same areas that underlie ones' own direct experiences, therefore allowing an automatic understanding of the cognitive/emotional states intrinsic to the complex emotion of regret in others.

So far, there was only behavioral evidence to suggest that the mere observation of a negative situation occurring to another individual evokes in the observer the same mental processes as those of the acting individual. These investigations assessed counterfactual reasoning in social contexts by comparing reported mental simulation*s* of actors, readers and observers of different situations all resolving negatively [8], [9]. These studies showed that actors (who made a decision and obtained a negative outcome) and readers (who read a story describing the actor's choice and outcome) produce different counterfactuals by focusing attention on different aspects of the situation [8]. However, when comparing actors' and observers' counterfactuals, these studies show that observers (who directly observed the actors' negative resolving situations) tend to mentally simulate alternative post-decisional solutions to those situations as actors themselves do [9]. These results suggest that, when faced with the negative outcome of another person's choices, individuals tend to react as they were personally involved in that situation.

Attending another's negative emotion, however, is a complex phenomenon that can elicit different and conflicting reactions in the beholder, as shown by two recent studies that have highlighted some of the several facets related to the understanding of others' emotions. These studies have addressed individuals' emotional responses arising from direct *social comparisons* [22], [23]. In Takahashi *et al.* [22], experimental contexts were defined a priori so as to elicit in the participants either the emotion of envy or gloating (*schadenfreude*). *f*MRI technique allowed to associate these emotions to the activation of dorsal ACC (envy) and of ventral striatum plus medial OFC (gloating), supporting the view that OFC activation is not specific for the processing of negative emotions. Bault *et al.*, [23], on the other hand, assessed the effects of one's own and others' previous outcomes on choice behavior in a gambling task. The authors observed that, when individuals played simultaneously on the same trials, the emotional (as assessed trough skin conductance response and heart-rate recording) and behavioral effects of envy and gloating (when the players made different choices) are stronger than the effects of regret and relief (when they made the same choices). In other words, these data show that, in a direct social confrontation, individuals' choice behavior is more strongly affected by the feelings of envy and gloating than by the emotions of regret or relief.

At a first glance, based on data from both these investigations, one might argue that the neural activations observed in the present study during “Other Plays” condition could relate to the emotion of gloating for the other player's misfortunes, rather than to regret. However, several considerations speak against this interpretation. Firstly, those studies were constructed so to elicit direct social comparisons between individuals by either manipulating participants' specific information or by having individuals playing on same trials. In the present study, the effect of possible social comparisons on the reported results was minimized. In fact, participants played on different trials and, particularly in study 1, the OP trials occurred immediately after the IP ones (direct social comparison) statistically only in 1 out of 32 trials. Additionally, outcomes producing the feelings of regret and relief were counterbalanced, thus further reducing the effect of gloating also when OP trials directly followed IP ones. Moreover, evidence that our results are not spoilt by the effects of gloating is represented by a lack of activation of the ventral striatum in OP task, which Takahashi *et al.* [22] indicate as its neural signature. Nonetheless, we do not reject the idea of possible different emotions, than regret, ultimately arising from the individual's awareness of someone else's regret. Still, our data clearly show that, in given contextual frames, e.g. when direct social comparison is minimized, and when individuals are aware of the process that leads to regret in others, observers neurally respond as they were directly involved in that situation. This neural process allows one to cognitively and emotionally reproduce the feeling experienced by a third person, thus leading to its automatic understanding.

A critical factor in the level of an individual's *shared* experience is her/his empathic aptitude. In this study, the behavioral results obtained on the BEES showed higher scores for females than males, particularly in study 2, suggesting that higher emphatic aptitude is associated with enhanced activation observed for females in vmPFC (see also [24]) and, only in study 2, in anterior insula (see Tables 4 and 5, Figure 4). Enhanced vmPFC activation for females during OP condition suggests that the engagement of the “resonant” mechanism in the regret network is particularly strong in emphatic individuals; insular activation, on the other hand, appears to be not related to regret *per se*, rather it can be more generally associated with the processing of emotional empathic responses, as also shown in previous studies [5], [7], [17].

On the whole, our data suggest that the emotional understanding of regret in others is specifically reflected by the activation of a subset of the regions involved in its direct, first-person, experience. Among these regions, vmPFC appears to be at the core of a counterfactual evaluation of the outcomes, updating the emotional valence of the obtained outcome with respect to that unobtained [12]. This evaluation results in the appropriate behavioral response associated with activity in ACC even when attending another's negative results. The finding of a resonance mapping system for the high-order experience of regret entails an important notion. In real social decisional contexts, one's own decisions and behaviors may be strongly influenced by interactive learning, i.e., learning from what other individuals experience as a result of their choices [25]. One might then wonder how such learning occurs, i.e. how the negative, regretful, outcomes of other individuals are coded in the decision-maker's brain. Does such a process involve the mere cold encoding of numerical quantities? The results of the present study show that this is not entirely the case. Rather, knowing the regretful outcomes of others' choices do lead to similar counterfactual comparisons and, via the reactivation of the same underlying cerebral regions, to the comprehension of the related emotional reactions, as experienced in a first-person perspective. This resonant emotion may represent a drive for behavioral reorganization even when attended in somebody else's experiences.

## Materials and Methods

### Study 1

#### Participants

Twenty-four healthy right-handed [26] monolingual native speakers of Italian (12 females [mean age = 25.75, s.d. = 2.18, range = 23.5–31.8] and 12 males [mean age = 25.34, s.d. = 2.90, range = 22–29.7]) participated in study 1. All participants had normal or corrected-to-normal visual acuity. None reported a history of psychiatric or neurological disorders, or current use of any psychoactive medications. They gave their written informed consent to the experimental procedure, which was approved by the Ethics Committee of San Raffaele Scientific Institute.

#### Task

The participants performed a classical gambling task [27]. In every trial, they were required to choose one of two gambles depicted as “wheels of fortune”, in which different probabilities of financial gain or loss are represented by the relative size of colored sectors of a circle. The gambles were then played and the results shown. Participants could thus evaluate not only the financial consequences of their decision, but also the outcome they might have obtained had they selected the alternative gamble. These evaluations gave them a sense of responsibility for their choices and determined a counterfactual reasoning, i.e., the main hallmarks of regret, when decisions produce relatively-negative outcome.

In the present investigation, there were two basic experimental conditions (see Figure 1). In the “*I play*” (IP) condition, participants were asked to choose one of two gambles, leading to a financial gain or loss for themselves. The gambles were shown for 5 s, during which they could evaluate them and make a decision. Next, the appearance of an asterisk in the centre of the screen prompted the participants to choose, by pressing one of two buttons on a keyboard with their right index or middle finger. The participants had 2 s to choose the gamble. In case they did not answer within this temporal window, they received an “out of time” message, and a new trial started. Once selected, the chosen gamble was highlighted by a white contour, and 3 s after the appearance of the asterisk the outcome of both gambles was shown for 3 s. In the “*Other plays*” (OP) condition, the participants were shown the same sequence of events (evaluation, decision and outcome, with the same timings) of the gamble played by an actor in a nearby room. In the OP condition, a small white square was shown along with the asterisk, either on its left or right side. The asterisk position indicated which gamble had just been chosen by the actor, and participants were asked to press the corresponding button. In order to focus their attention on the gamble-results in both IP and OP conditions, and to assess the participants' understanding of the other players' emotional state at outcome evaluation, after outcome presentation the participants had to indicate whether they were satisfied with their own result (IP) or whether the actor was satisfied with her result (OP), by pressing one of two buttons (left: yes, right: no; 3 s).

As an explicit-baseline, two further conditions were used: in the “*I follow*” (IF) and “*Other follows*” (OF) conditions, participants were informed that the computer would randomly choose one of the gambles, for themselves or for the other player, respectively. In these conditions, the decision-period lasted 2 s. Like in the OP condition, the decision made by the computer was signaled by a small white square appearing along with the asterisk, and participants were simply asked to press the corresponding left/right button. These trials still resulted in financial gains or losses for the participants or the actor, yet enabled us to control for the feeling of responsibility for the gamble choice, which is a crucial determinant of the emotion of regret.

Each trial started with a specific instruction indicating the condition type (1 s), which remained at the bottom of the screen throughout the trial length. All instructions were presented in Italian.

#### Gambles structure

The participants underwent a total of 256 trials (64 for each experimental condition). The complete list of trials was predetermined and identical for all the participants. In each gamble, the 4 possible outcomes resulted from paired combinations of 200, 50, −50 and −200 (arbitrary units), associated with 8 different levels of probability (30-70, 35-65, 40-60, 45-55, 55-45, 60-40, 65-35, 70-30). Thus, the possible combinations of wins and losses gave four potential levels of regret (−100, −150, −250 and −400) and relief (100, 150, 250 and 400). The possible combinations of payoffs and levels of probability were equally balanced across all experimental conditions. In each trial, payoffs and probabilities were associated so that a) one of the gambles was riskier than the other, and b) the difference between the gambles was minimized with regard to the expected-value (i.e., the sum of the probability of the two possible gamble outcomes, each multiplied by the corresponding outcome value). In order to compare the effects of different experienced *vs*. attended amounts of regret, it was crucial to outbalance the number of events of interest across the different experimental conditions. Therefore, unbeknownst to the participants, the list of stimuli was arranged so that in OP, IF and OF conditions every single trial resulted in a pre-determined pair of outcomes (and thus in a pre-specified amount of either regret or relief in the OP condition). In order to make sure that the number of regret and relief events balanced out in the IP task (where we had no control on the participant's choice), every trial was pre-determined to necessarily result in a variable amount of either regret or relief by means of a feedback-routine. For every task, the obtained “regret” and “relief” trials were then assigned to the different functional runs so to obtain a variable proportion of events of regret and relief. Crucially, to preserve a most realistic probabilistic scenario, in all conditions we ensured that, across trials, the least probable gamble outcomes would occur in a proportion equal or inferior to 50% (OP = 47%; IF and OF = 50%; IP = 42%). In fact, as confirmed by the post-scanning debriefing, all participants were unaware of the experimental control on the probabilistic occurrence of wins and losses.

#### Instructions and procedure

The participants underwent a training session and were introduced to the same unknown female actor before the beginning of the study. Moreover, they were informed that both their and the actor's performance in IP/IF and OP/OF tasks, respectively, would have resulted in a financial gain or loss with respect to an initial endowment. Importantly, to constrain a competitive attitude towards the actor's performance, participants were explicitly informed that their potential gains/losses were completely independent of those of the other player. Additionally, when introducing the actor to the participants, the actor's personal profile was purposely kept very low. The participants were informed about their cumulative earnings only outside the scanner, after the functional acquisition.

The study was composed of 8 functional runs. Every run comprised 32 trials (8 for each experimental condition). These were randomly assigned to 8 blocks, each of which contained 4 consecutive trials of the same condition. The order of the functional runs, of the blocks within each run and of the trials within each block were randomized across participants. Null events were also included in every run, to allow estimation of low-level baseline brain activity. In order to desynchronize the timings of event-types with respect to the acquisition of single slices within functional volumes, interstimulus intervals (ISI) between successive trials were presented in different (“jittered”) durations across trials (1350, 1950, and 2550 s, in proportion of 4∶2∶1; [28]).

Visual stimuli were viewed via a back-projection screen located in front of the scanner and a mirror placed on the head-coil. The software Presentation 11.0 (Neurobehavioral systems, Albany, CA, http://www.neurobs.com) was used both for stimulus presentation and participants' answers recording.

After the scanning, participants were asked to report their personal impressions about the task. Then, they completed an Italian version [14] of the Balanced Emotional Empathy Scale (BEES; [15]), a 30-item questionnaire on emphatic abilities designed to measure individual tendency to empathize with others' emotional experiences (i.e., emotional empathy).

### Study 2: Differences with respect to study 1

#### Participants

Twenty-four healthy right-handed [26] monolingual native speakers of Italian (12 females [mean age = 20.28, s.d. = 1.16, range = 19–23] and 12 males [mean age = 22.86, s.d. = 3.26, range = 19–30]) participated in study 2.

#### Task

Three main differences distinguished study 2 from study 1 with regard to the task. Firstly, the emotional component of post-outcome judgment was replaced by a “cold” appraisal of the obtained outcome. Namely, instead of providing a satisfaction-judgment, the participants were required to indicate whether the gamble outcome was a win or a loss. Second, in study 2 participants' response was required in all four conditions (IP, OP, IF, OF) and only on 10% of the trials. Finally, the length of the evaluation phase (gambles presentation) was identical in all four conditions (4.5 s).

#### Gambles structure

Different from study 1, in each gamble the 4 possible outcomes resulted from paired combinations of 200, 50, −50 and −200 (arbitrary units), associated with only 3 different levels of probability (25-75, 50-50, 75-25). However, the possible combinations of wins and losses still gave four potential levels of regret (−100, −150, −250 and −400) and relief (100, 150, 250 and 400).

#### Instructions and procedure

All participants underwent a training session, and were introduced to an unknown actor. In study 2, half of them (50% females and 50% males) were presented to a female actor and the other half to a male actor.

#### fMRI data acquisition and statistical analysis

Anatomical T1-weighted and functional T2*-weighted MR images were acquired with a 3 Tesla Philips Achieva scanner (Philips Medical Systems, Best, NL), using an 8-channels Sense head coil (sense reduction factor = 2). Functional images were acquired using a T2*-weighted gradient-echo, echo-planar (EPI) pulse sequence (38 interleaved coronal slices covering the whole brain, TR = 2200 ms, TE = 30 ms, flip-angle = 85 degrees, FOV = 240 mm×240 mm, inter-slice gap = 0.5 mm, slice thickness = 4 mm, in-plane resolution 2.5 mm×2.5 mm). Each scanning sequence comprised 215 sequential volumes. Immediately after the functional scanning a high-resolution T1-weighted anatomical scan (150 slices, TR = 600 ms, TE = 20 ms, slice thickness = 1 mm, in-plane resolution 1 mm×1 mm) was acquired for each participants.

Image pre-processing and statistical analysis were performed using SPM5 (Wellcome Department of Cognitive Neurology, http://www.fil.ion.ucl.ac.uk/spm), implemented in Matlab v7.4 (Mathworks, Inc., Sherborn, MA) [29]. The first 5 volumes of each participant were discarded to allow for T1 equilibration effects. All volumes were then spatially realigned [30] to the first volume of the first session to correct for between-scan motion and unwarped [31], and a mean-image from the realigned volumes was created. This image was spatially normalized to the Montreal Neurological Institute 305 (MNI305) brain template using a 12-parameter affine normalization and 16 nonlinear iterations with 7×9×7 basis functions [32]. The derived spatial transformations were then applied to the realigned-and-unwarped T2*-weighted volumes, that were resampled in 2×2×2-mm voxels after normalization. All functional volumes were then spatially smoothed with an 8-mm full-width half-maximum (FWHM) isotropic Gaussian kernel to compensate for residual between-subject variability after spatial normalization, and globally scaled to 100. The resulting time series across each voxel were then high-pass filtered to 1/128 Hz, and serial autocorrelations were modeled as an Auto-Regressive AR(1) process.

Statistical maps were generated using a random-effect model, implemented in a 2-levels procedure [33].

At the first level, two sets of analyses were performed. Firstly, outcome trials were partitioned according to the 4 conditions (IP, IF, OP, OF) which were separately modeled as mini-epoch lasting 3 s. For each of the 4 conditions, one additional regressor modeled a linear parametric modulation of the outcome-related activity by the degree of objective amount of *regret/relief* (computed as the difference between the actual and unobtained outcomes). In line with Coricelli *et al.*'s [12] procedure , in a second analysis we modeled a linear parametric modulation by the degree of *satisfaction/disappointment*, i.e., the amount of discrepancy between the obtained and unobtained outcomes in the *chosen* gamble only. All the within-trials events other than the outcomes, as well as those trials in which a wrong response or no response was given, were modeled in a single regressor of no interest. Regressors modeling events were convolved with a canonical Haemodynamic Response Function (HRF), and parameter estimates for all regressors were obtained at each voxel by maximum-likelihood estimation. Contrasts of parameter estimates were then calculated to produce “contrast images” for each contrast of interest (“IP *minus* IF” and “OP *minus* OF” for both regret- and disappointment-related parametric regressors).

At the second (group) level, these two types of contrast-image were used to perform separate parametric (i.e., dependent on the degree of either regret or disappointment) analyses. Furthermore, since we aimed at investigating also potential gender effects on “mirror-like” cerebral activity, the 1^st^-level contrast images for “IP *minus* IF” and “OP *minus* OF” for male and female participants were entered into a 2×2 [perspective (“IP *vs*. OP”) by gender (female *vs*. male)] factorial design with sphericity-correction for repeated measures [34]. Based on a-priori hypotheses from a previous study [12], the resulting statistical maps were thresholded at p<0.001 uncorrected for multiple comparisons, and only clusters larger than 5 voxels were reported.

In order to assess common effects across IP and OP tasks, we carried out a conjunction analysis on the IP (*minus* IF) and OP (*minus* OF) statistical maps for both the disappointment- and the regret-related parametric effects. This analysis was done using an inclusive masking procedure, in which the statistical maps for OP conditions were inclusively masked by those for the IP condition. Finally, direct comparisons were performed to assess perspective- and gender-effects on condition-related cerebral activity in both analyses. The resulting statistical maps were thresholded at p<0.001 uncorrected for multiple comparisons and, in order to ensure that the observed activations did not result from relative deactivations, they were inclusively masked at p<0.05 uncorrected by those associated with the conditions of interest *minus* the baseline task.

The location of the activation foci in terms of Brodmann Areas (BAs) was determined in the stereotaxic space of Talairach and Tournoux [35] after correcting for differences between the latter and the MNI coordinate systems by means of a nonlinear transformation (see http://www.mrc-cbu.cam.ac.uk/Imaging/Common/mnispace.shtml). Those cerebral regions for which maps are provided were also localized with reference to cytoarchitectonical probabilistic maps of the human brain, using the SPM-Anatomy toolbox [36].

## Supporting Information

Text S1 Behavioral results in study 1
(0.03 MB DOC)

Table S1 Cerebral activations in IP condition in study 1
(0.08 MB DOC)

Table S2 Cerebral activations in OP condition in study 1
(0.06 MB DOC)

Table S3 Cerebral activations resulting from the direct comparisons IP versus OP conditions in study 1
(0.05 MB DOC)

Figure S1 Cerebral activations in the IP (minus baseline) and OP (minus baseline) conditions in study 1
(0.92 MB TIF)
