# Supplementary material for: Understanding Others' Regret: A fMRI Study
Source: PLoS One. 2009 Oct 14;4(10):e7402. doi: 10.1371/journal.pone.0007402 (PMC2756584; doi:10.1371/journal.pone.0007402)
Supplement: Text S1 — Behavioral results in study 1 (0.03 MB DOC) [file pone.0007402.s001.doc]

In the conditions IF, OF and OP subjects were to provide a response that indicated that they had paid attention to the decision made either by the computer (IF, OF) or by the other player (OP). Namely, subjects were asked to press a button corresponding to the position of a small square displayed either on the left or right half of the screen, and indicating which gamble had just been chosen by the actor or the computer. No significant differences were observed in response-accuracy among OP (mean = 99.54, standard-deviation [s.d.] = 0.85), IF (mean = 99.34, s.d. = 1.12) and OF conditions (mean = 99.47, s.d. = 0.88) (Kolmogorov-Smirnov test for normality: d = 0.33, Lilliefors-corrected [1] p < 0.01; non-parametric Friedman ANOVA, N = 24, Chi-Square (2) = 0.36, p = 0.83), indicating that subjects paid attention to all conditions, regardless of their active involvement in the decision process.

In order to make sure that the participants’ emotional reaction was consistent with the actual counterfactual comparison between the obtained and unobtained outcomes, they had to indicate whether they were satisfied with their own decision (IP). Moreover, to ascertain that participants were also aware of the other player’s emotional response to the outcome, in the OP condition they had to indicate whether, in their opinion, the other player was satisfied with her/his decision. Results indicated that there was a high consistency between satisfaction judgments and the actual outcomes in both IP (mean = 95.96, s.d. = 6.23) and OP (mean = 95.31, s.d. = 4.58) conditions (Kolmogorov-Smirnov test for normality: d = 0.42, Lilliefors-corrected [1] *p* < 0.01; non parametric Wilcoxon-matched-pairs-test, N = 24, *T* = 27, *Z* = 0.53, *p* = 0.59), indicating that the evoked emotions at outcome conformed with the gamble results.

1. Lilliefors HW (1967) On the Kolmogorov– Smirnov test for normality with mean and variance unknown. J Am Stat Assoc: 399-402.
