# Supplementary material for: Understanding Others' Regret: A fMRI Study
Source: PLoS One. 2009 Oct 14;4(10):e7402. doi: 10.1371/journal.pone.0007402 (PMC2756584; doi:10.1371/journal.pone.0007402)
Supplement: Table S1 — Cerebral activations in IP condition in study 1 (0.08 MB DOC) [file pone.0007402.s002.doc]

| H | Anatomical region (BA) | x | MNI  y | z | Z-score |
| --- | --- | --- | --- | --- | --- |
|  | **IP *minus* IF** |  |  |  |  |
|  |  |  |  |  |  |
| L | vmPFC (11) | -18 | 38 | -14 | 3.82 |
|  | vmPFC (11) | -14 | 48 | -14 | 3.75 |
| R | vmPFC (11) | 10 | 48 | -8 | 3.26 |
| L | Anterior cingulate cortex (24/32) | -10 | 44 | 16 | 3.60 |
|  | Superior medial gyrus (32/10) | -8 | 50 | 8 | 4.88 |
| L/R | Anterior cingulate cortex (24) | 0 | 34 | 14 | 4.28 |
| L/R | Anterior cingulate cortex (24/32) | -2 | 34 | 26 | 3.21 |
| L | Middle cingulate cortex (24/6) | -12 | 4 | 42 | 5.24 |
| L/R | Middle cingulate cortex (24) | 0 | 0 | 30 | 3.69 |
| L/R | SMA (6) | 4 | -16 | 64 | 4.45 |
|  |  |  |  |  |  |
| L/R | Middle cingulate cortex (6) | 0 | -2 | 48 | 4.16 |
|  |  |  |  |  |  |
| L/R | Middle cingulate cortex (6) | 2 | -16 | 50 | 3.86 |
|  |  |  |  |  |  |
| L/R | Posterior cingulate cortex (23) | 2 | -42 | 26 | 3.58 |
| L | Amygdala | -18 | 2 | -18 | 4.11 |
|  | Insula lobe | -24 | 12 | -18 | 4.23 |
|  | Medial temporal pole (38) | -30 | 8 | -20 | 4.13 |
| L | Temporal pole (38) | -52 | 12 | -18 | 4.32 |
| L | Amygdala/Hippocampus | -34 | -8 | -30 | 4.62 |
| L | Hippocampus | -34 | -32 | -10 | 3.70 |
| R | Hippocampus/ Amygdala | 32 | -8 | -30 | 4.51 |
|  |  |  |  |  |  |
| R | Hippocampus | 18 | -26 | -14 | 4.24 |
| R | Hippocampus | 18 | -36 | 4 |  |
| R | Middle temporal gyrus (21) | 58 | -10 | -18 | 3.46 |
| L | Postcentral gyrus (1/4a) | -56 | -12 | 44 | 3.94 |
|  | Postcentral gyrus (1/3b) | -60 | -10 | 38 | 3.41 |
| L | Postcentral gyrus (OP4) | -64 | -10 | 20 | 3.43 |
| R | IPL/Postcentral gyrus (2) | 42 | -40 | 56 | 3.21 |
|  | Postcentral gyrus (2/1) | 48 | -34 | 56 | 3.19 |
| L | Cerebellum (VI) | -30 | -44 | -26 | 5.41 |
| L | Cerebellum (IV-V) | -20 | -36 | -24 | 3.69 |
| L | Cerebellum (Crus1) | -38 | -74 | -24 | 3.35 |
| R | Cerebellum (VI) | 22 | -54 | -22 | 5.04 |
| R | Cerebellum (Crus1) | 38 | -46 | -40 | 3.65 |
| L | Cerebellum (VIII) | -4 | -64 | -34 | 3.88 |
| R | Cerebellum (VIII) | 8 | -60 | -34 | 4.10 |
| L/R | Cerebellar vermis (8) | -2 | -60 | -26 | 4.95 |

H = Hemisphere, L = Left, R = Right, BA = estimated Brodmann Area, vmPFC = ventromedial Prefrontal Cortex, SMA = Supplementary Motor Area, IPL = Inferior Parietal Lobule.
