# Supplementary material for: Understanding Others' Regret: A fMRI Study
Source: PLoS One. 2009 Oct 14;4(10):e7402. doi: 10.1371/journal.pone.0007402 (PMC2756584; doi:10.1371/journal.pone.0007402)
Supplement: Table S2 — Cerebral activations in OP condition in study 1 (0.06 MB DOC) [file pone.0007402.s003.doc]

| H | Anatomical region (BA) | x | MNI  y | z | Z-score |
| --- | --- | --- | --- | --- | --- |
|  | **OP *minus* OF** |  |  |  |  |
|  |  |  |  |  |  |
| L | vmPFC (11) | -10 | 46 | -18 | 5.34 |
| R | vmPFC (11) | 14 | 46 | -16 | 4.39 |
| L | Anterior cingulate cortex (24/32) | 2 | 40 | 10 | 3.10 |
| L | SMA (6) | -6 | -10 | 56 | 4.15 |
| L/R | Middle cingulate cortex (6/23) | 0 | -10 | 40 | 3.76 |
| R | SMA (6) | 4 | -14 | 52 | 3.39 |
| L | Amygdala | -26 | 2 | -20 | 3.45 |
| L | Hippocampus | -22 | -14 | -24 | 4.11 |
| L | Hippocampus | -32 | -24 | -22 | 3.52 |
| L | Hippocampus | -30 | -34 | -12 | 4.52 |
| R | Hippocampus | 32 | -12 | -32 | 3.59 |
|  | Hippocampus | 30 | -14 | -28 | 3.56 |
| R | Hippocampus* | 38 | -30 | -16 | 4.49 |
| R | Hippocampus* | 36 | -26 | -14 | 4.27 |
| R | Hippocampus | 28 | -34 | -4 | 3.29 |
| L | Medial temporal pole (20/38) | -38 | 12 | -34 | 3.94 |
| R | Medial temporal pole (38) | 48 | 18 | -34 | 3.73 |
| R | Middle temporal gyrus (21) | 58 | -8 | -22 | 3.82 |
| L | Postcentral gyrus (1/3b) | -22 | -38 | 68 | 4.82 |
|  | Postcentral gyrus (1/3b) | -30 | -34 | 66 | 4.12 |
|  | Postcentral gyrus (4a/6) | -20 | -26 | 68 | 3.95 |
|  | Postcentral gyrus (4a/6) | -18 | -30 | 68 | 3.83 |
| R | Postcentral gyrus (3a) | 42 | -8 | 28 | 3.58 |
|  | Postcentral gyrus (3a) | 38 | -12 | 30 | 3.14 |
| R | Postcentral gyrus (2) | 26 | -38 | 52 | 3.38 |

H = Hemisphere, L = Left, R = Right, BA = estimated Brodmann Area, vmPFC = ventromedial Prefrontal Cortex, SMA = Supplementary Motor Area.
