# Supplementary material for: Understanding Others' Regret: A fMRI Study
Source: PLoS One. 2009 Oct 14;4(10):e7402. doi: 10.1371/journal.pone.0007402 (PMC2756584; doi:10.1371/journal.pone.0007402)
Supplement: Table S3 — Cerebral activations resulting from the direct comparisons IP versus OP conditions in study 1 (0.05 MB DOC) [file pone.0007402.s004.doc]

| H | Anatomical region (BA) |  | MNI |  | Z-score |
| --- | --- | --- | --- | --- | --- |
|  |  | x | y | z |  |
|  | **a. IP (*minus* IF) *vs*. OP (*minus* OF)** |  |  |  |  |
|  |  |  |  |  |  |
| L/R | Posterior cingulate cortex (26) | 0 | -40 | 22 | 3.71 |
| L | Medial superior frontal gyrus (9) | -16 | 48 | 34 | 4.38 |
| R | Medial superior frontal gyrus (9) | 16 | 54 | 38 | 4.07 |
| L/R | Cerebellar vermis | 0 | -62 | -34 | 4.49 |
| L | Cerebellum (VI) | -18 | -82 | -18 | 3.54 |
| R | Cerebellum (VI) | 24 | -72 | -18 | 4.47 |
|  |  |  |  |  |  |
|  | **b. OP (*minus* OF) *vs*. IP (*minus* IF)** |  |  |  |  |
|  |  |  |  |  |  |
| L | vmPFC (11) | -12 | 46 | -16 | 3.18 |
| R | vmPFC (11) | 10 | 46 | -18 | 3.10 |
| R | Postcentral gyrus (3b) | 30 | -34 | 64 | 3.85 |
|  | Postcentral gyrus (3b) | 22 | -38 | 68 | 3.73 |
| L | Hippocampus | -36 | -26 | -12 | 3.34 |
| R | Hippocampu | 30 | -24 | -20 | 4.00 |

H = Hemisphere, L = Left, R = Right, BA = estimated Brodmann Area, vmPFC = ventromedial Prefrontal Cortex.
