# Supplementary figures and images for: Understanding Others' Regret: A fMRI Study
Source: PLoS One. 2009 Oct 14;4(10):e7402. doi: 10.1371/journal.pone.0007402 (PMC2756584; doi:10.1371/journal.pone.0007402)

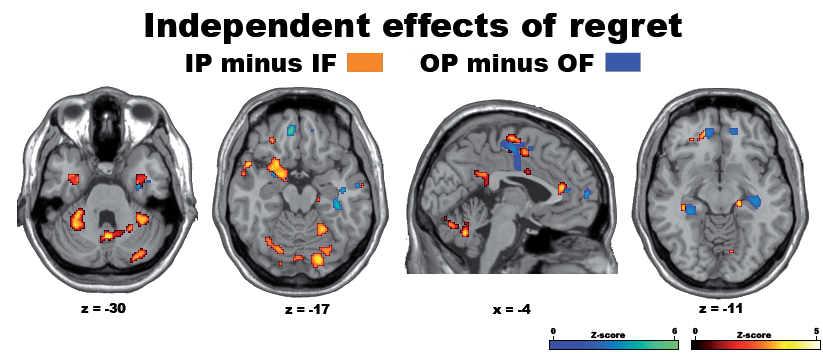

Supplement: Figure S1 — Cerebral activations in the IP (minus baseline) and OP (minus baseline) conditions in study 1 (0.92 MB TIF) [file pone.0007402.s005.tif]
